# Choroidal Thickness Measurements in the Case of Diabetic Macular Edema. Comment on Amjad et al. Choroidal Thickness in Different Patterns of Diabetic Macular Edema. *J. Clin. Med.* 2022, *11*, 6169

**DOI:** 10.3390/jcm12082874

**Published:** 2023-04-14

**Authors:** Andrea Valerio Marino, Martina De Luca, Ludovica Reda, Marco Gioia

**Affiliations:** Department of Medicine, Surgery and Dentistry “Scuola Medica Salernitana”, University of Salerno, 84100 Baronissi, Italy

We read with great interest the article by Amjad R. et al. on “Choroidal Thickness in Different Patterns of Diabetic Macular Edema” [1].

We appreciated this paper because the impact of diabetic macular edema (DME) is one of the most common causes of visual loss.

These types of studies are always very interesting, but we would like to make some comments.

In the literature, ChT measurements have been used to explain several conditions, [2,3,4], and it has been clearly shown that the choroid is thicker in smaller eyes and thinner in bigger ones. Unfortunately, in this paper, the axial length was not assessed; therefore, different types of DME patients cannot be compared, and resulting thickness differences could be affected by bias. We are aware that axial length measurements need some corrections, but they are precise enough for such an aim [5,6].

However, we are a bit concerned about the reliability of (ChT) measurements in the case of macular edema because, in these patients, exudation in the intraretinal space makes foveal recognition difficult due to the disappearance of the foveal depression, and in results, it is difficult to identify the right point to place the marker for measurement [7]. Moreover, the authors’ objective was to measure the subfoveal (ChT) on pixel/micron-scale images, but (ChT) evaluation should be performed on images in the micron scale and with a line perpendicular to the RPE-Bruch complex.

Finally, we question the selection of healthy eyes because the authors used 57 eyes from 30 patients and did not select both eyes from every healthy patient.
